# Fine-Scale Modeling of Individual Exposures to Ambient PM_2.5_, EC, NO_x_, CO for the Coronary Artery Disease and Environmental Exposure (CADEE) Study

**DOI:** 10.3390/atmos11010065

**Published:** 2020-01-03

**Authors:** Michael Breen, Shih Ying Chang, Miyuki Breen, Yadong Xu, Vlad Isakov, Sarav Arunachalam, Martha Sue Carraway, Robert Devlin

**Affiliations:** 1 Center for Public Health and Environmental Assessment, U.S. Environmental Protection Agency, Research Triangle Park, NC 27711, USA; 2 Institute for the Environment, University of North Carolina at Chapel Hill, Chapel Hill, NC 27517, USA; 3 Center for Public Health and Environmental Assessment, ORISE/U.S. Environmental Protection Agency, Chapel Hill, NC 27514, USA; 4 ORAU/U.S. Environmental Protection Agency, Research Triangle Park, NC 27711, USA; 5 Center for Measurements and Modeling, U.S. Environmental Protection Agency, Research Triangle Park, NC 27711, USA; 6 Department of Medicine, Pulmonary and Critical Care Medicine, Durham VA Medical Center, Durham, NC 27705 USA; 7 Center for Public Health and Environmental Assessment, U.S. Environmental Protection Agency, Chapel Hill, NC 27514, USA

**Keywords:** air pollution, exposure modeling, particulate matter, gaseous pollutants, building infiltration modeling

## Abstract

Air pollution epidemiological studies often use outdoor concentrations from central-site monitors as exposure surrogates, which can induce measurement error. The goal of this study was to improve exposure assessments of ambient fine particulate matter (PM_2.5_), elemental carbon (EC), nitrogen oxides (NO_x_), and carbon monoxide (CO) for a repeated measurements study with 15 individuals with coronary artery disease in central North Carolina called the Coronary Artery Disease and Environmental Exposure (CADEE) Study. We developed a fine-scale exposure modeling approach to determine five tiers of individual-level exposure metrics for PM_2.5_, EC, NO_x_, CO using outdoor concentrations, on-road vehicle emissions, weather, home building characteristics, time-locations, and time-activities. We linked an urban-scale air quality model, residential air exchange rate model, building infiltration model, global positioning system (GPS)-based microenvironment model, and accelerometer-based inhaled ventilation model to determine residential outdoor concentrations (C_out_home_, Tier 1), residential indoor concentrations (C_in_home_, Tier 2), personal outdoor concentrations (C_out_personal_, Tier 3), exposures (E, Tier 4), and inhaled doses (D, Tier 5). We applied the fine-scale exposure model to determine daily 24-h average PM_2.5_, EC, NO_x_, CO exposure metrics (Tiers 1–5) for 720 participant-days across the 25 months of CADEE. Daily modeled metrics showed considerable temporal and home-to-home variability of C_out_home_ and C_in_home_ (Tiers 1–2) and person-to-person variability of C_out_personal_, E, and D (Tiers 3–5). Our study demonstrates the ability to apply an urban-scale air quality model with an individual-level exposure model to determine multiple tiers of exposure metrics for an epidemiological study, in support of improving health risk assessments.

## 1. Introduction

Epidemiological studies have found associations between exposure to ambient (i.e., outdoor-generated) fine particulate matter (PM_2.5_; particulate matter ≤ 2.5μm in aerodynamic diameter) and its component elemental carbon (EC), nitrogen oxides (NO_x_), and carbon monoxide (CO) and indices of acute respiratory and cardiovascular morbidity and mortality [1–4]. Most of these studies used central-site measurements of these air pollutants as exposure surrogates due to cost and participant burden of using indoor or personal air pollution monitoring devices. While these exposure surrogates are designed for studies where central site monitor is representative for the entire study domain, they might have limitations in urban-scale studies where air pollution concentrations can be highly elevated near transportation sources such as highways, railroads, or airports. Specifically, these exposure surrogates do not account for (1) fine-scale spatial and temporal variability of on-road vehicle emissions and dispersion, (2) building-to-building and temporal variability of indoor infiltration (i.e., attenuation) of ambient air pollutants, (3) person-to-person and temporal variability of time spent in different indoor and outdoor locations, and (4) variability of respiratory inhalation (i.e., inhaled dose) from time spent at various physical activity levels. Differences between exposure surrogates, such as central-site measurements, and true exposures contribute to exposure measurement error. Depending on the epidemiological study design, these errors can add bias or uncertainty in health effect estimates [5–6]. The significance of this issue was highlighted in several reports by the National Research Council and National Academies of Sciences [7–10]. To address the recommendations of these reports, we developed the Exposure Model for Individuals (EMI), which can help reduce measurement error and improve health effect estimation [11–15]. This study describes the application of EMI for ambient PM_2.5_, EC, NO_x_, and CO in the Coronary Artery Disease and Environmental Exposure study (CADEE) [16].

The goal of CADEE is to examine ambient air pollutant exposures and cardiovascular and hematologic effects in adults with coronary artery disease living in central North Carolina (NC). Using ozone measurements from two fixed-site air monitors, significant associations were previously found between daily ambient ozone concentrations and various acute (maximum lag of 5 days) adverse effects: (1) altered endothelial function, (2) increased blood levels of inflammatory markers: neutrophils, monocytes, and interleukin-6, and (3) increased blood levels of factors attributed to fibrinolysis: tissue plasminogen factor and plasminogen activator inhibitor-1 [16]. In this study, we applied EMI for a subsequent epidemiological analysis to address the possible limitation of using outdoor air pollutant concentrations from fixed-site monitors as exposure surrogates in CADEE.

The EMI predicts multiple tiers of individual-level exposure metrics for actual participants in epidemiological studies using outdoor concentrations, questionnaires, weather, and time-activity information [11]. We previously developed and applied EMI for an epidemiological study called the Diabetes and the Environment Panel Study (DEPS) [11–12]. In DEPS, we used a residential air exchange rate (AER) model, building infiltration model, and microenvironment-based exposure model to predict residential indoor concentrations and personal exposures for ambient PM_2.5_.

For CADEE, we extended EMI to develop a refined exposure modeling approach that includes six additional capabilities. First, the exposure model includes four pollutants (PM_2.5_, EC, NO_x_, CO), whereas DEPS included only PM_2.5_ [12]. Second, we used a previously developed urban-scale air quality model (AQM) to determine background, on-road, and total concentrations of each pollutant [17]. Third, we used a previously evaluated global positioning system (GPS)-based microenvironment (ME) classification model called MicroTrac to determine time-spent in different ME, whereas for DEPS we used time-location diary information [18]. Finally, an accelerometer-based ventilation model called VTrac was developed and applied to predict inhaled dose from physical activity information.

Before applying EMI for epidemiological studies with limited exposure data, we previously calibrated and evaluated EMI with extensive exposure data from field studies to reduce model uncertainty. We used measurement data from multiple field studies to evaluate the residential AER model, infiltration model, and GPS-based MicroTrac model [11,12,14,15,18]. Using a cross-validation, we compared individual predictions with 591 daily measurements from 31 homes and participants in central NC, which is the same location as CADEE. Median absolute differences were 20% (2.0 μg/m^3^) for home indoor concentrations and 20% (1.8 μg/m^3^) for personal exposures for ambient PM_2.5_ [11].

In this paper, we develop ambient PM_2.5_, EC, NO_x_, CO exposure metrics for CADEE. We used outdoor concentrations and on-road vehicle emission factors as inputs for the AQM, and used housing characteristics, weather, time-locations from GPS loggers, and time-activities from accelerometers as inputs for EMI. We will first describe the CADEE design, and then describe the AQM and EMI algorithms, and the development of multiple tiers of daily exposure metrics for each study participant.

## 2. Materials and Methods

### 2.1 CADEE Design

The CADEE study was designed to examine the relationship between exposures to different air pollutants and various indices of acute cardiovascular and hematologic effects in a cohort of adults with coronary artery disease. A previous publication describes the study design and clinical measurements [16]. Briefly, the study included 15 non-smoking adult participants that had undergone a cardiac catheterization at Duke University Hospital and resided in central NC. Each participant visited the U.S. Environmental Protection Agency (EPA) Human Studies Facility (HSF) in Chapel Hill, NC at 8 am (± 1.5 h) for two consecutive weekdays for up to 10 weeks between May 2012 and April 2014. On the first day, the participant was outfitted with a hip-mounted accelerometer (model Actical; Respironics Inc., Murrysville, PA, USA) and provided a GPS data logger (model BT-Q1000XT; Qstartz International, Taipei, Taiwan), which they carried for the next 24 h. Various clinical measurements were collected at baseline and the following day to yield a total of 120 participant-days of data. Written informed consent was given by all participants prior to enrollment, and the study was approved by the Duke University Institutional Review Board, the University of North Carolina at Chapel Hill Institutional Review Board, and the EPA Human Protocols Office.

Input data for EMI were obtained from the participants for their home building characteristics, time-locations, and time-activities. Daily questionnaires were used to collect occupant behavior related to building operation, including indoor temperature, open windows and doors, and operating window fans. The GPS and accelerometer data loggers were used to collect continuous participant locations and physical activity intensities; respectively.

Before each 24 h deployment of the GPS data logger, the GPS memory was cleared using QTravel software (version 1.2; Qstartz International, Taipei, Taiwan) and the battery was fully charged. The GPS was programmed to sample every 5 sec and to collect the date, time, position (latitude, longitude), speed, number of satellites used, and position dilution of precision (dimensionless value ≥ 1 that indicates accuracy of GPS position due to the satellite geometry) [18]. The sampled data were stored in the GPS memory during the 24 h sampling period, and then downloaded and stored in a text file for the MicroTrac model described below.

Before each 24 h deployment of the accelerometer data logger, the accelerometer memory was cleared using Actical software (version 3.0; Respironics Inc., Murrysville, PA, USA). The accelerometer was programmed for 1-sec epochs and to collect the date, time, activity counts (value that indicates intensity of motion). The sampled data were stored in the accelerometer memory during the 24 h sampling period, and then downloaded and stored in a text file for the VTrac model described below.

### 2.2 Tiers of modeled exposure metrics

**Figure 1.**
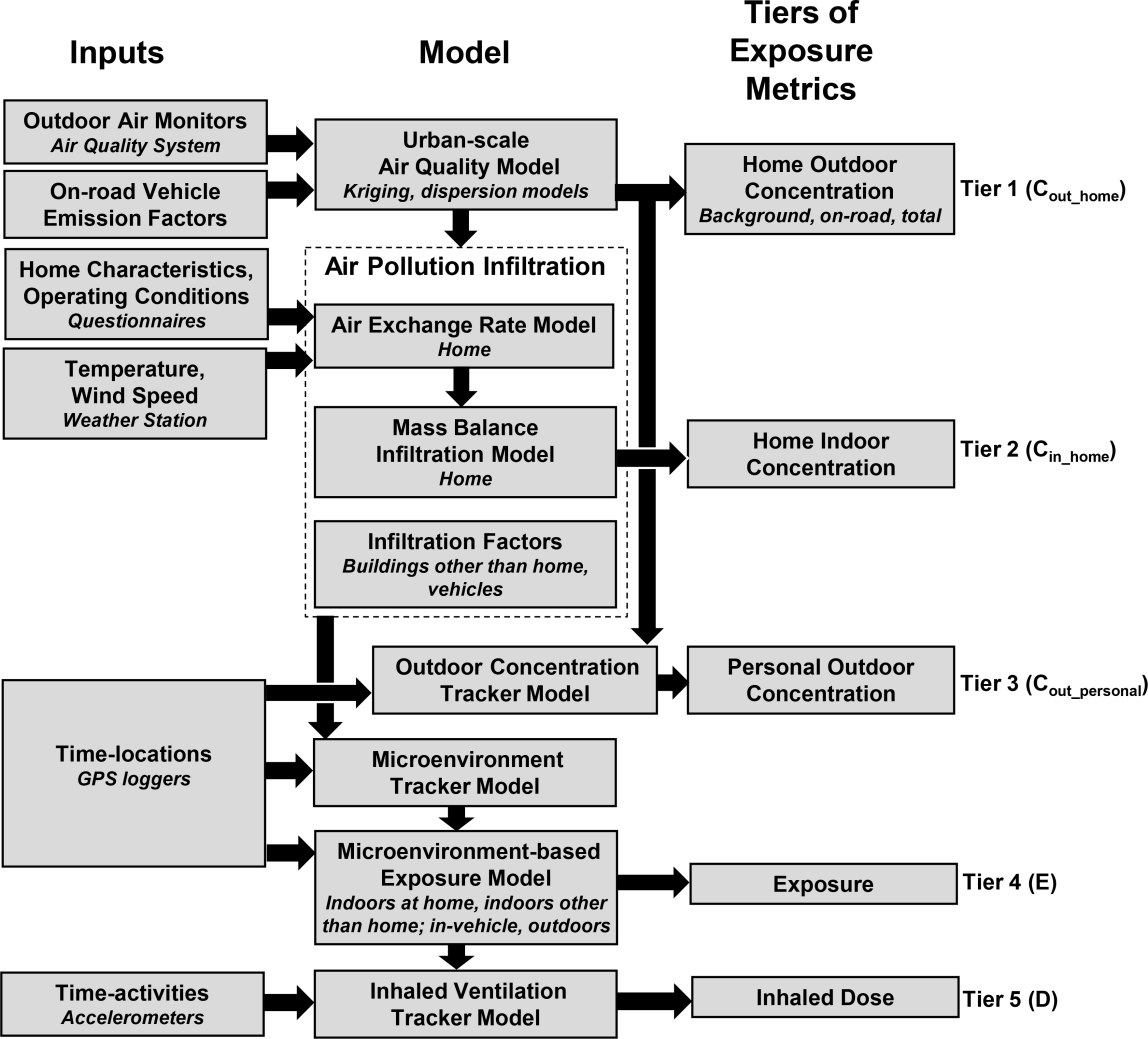
Conceptual model of EMI to predict five tiers of individual-level exposure metrics for ambient PM_2.5_, EC, NO_x_, and CO. Tiers 1–2 (C_out_home_ – outdoor concentration, C_in_home_ – indoor concentration) are related to homes, and Tiers 3–5 (C_out_personal_ – personal outdoor concentration, E – exposure, D – inhaled dose) are related to participants.

We modeled five tiers of daily exposure metrics for ambient PM_2.5_, EC, NO_x_, CO for 15 study participants and their homes (Figure 1). The five tiers, which have increasing levels of complexity and information needs, include: (Tier 1) home outdoor concentrations; (Tier 2) home indoor concentrations; (Tier 3) personal outdoor concentrations; (Tier 4) exposures; (Tier 5) inhaled doses. Each tier is separated into contributions from background, on-road emissions and total. For each participant, 24-h average (8 am to 8 am) exposure metrics were modeled on the days with clinical measurements, and on the five days before clinical visits to yield a total of 720 participant-days. The modeling and subsequent analysis were implemented using MATLAB software (version R2015a, Mathworks, Natick, MA, USA).

#### 2.2.1 Home outdoor concentrations (Tier 1)

For Tier 1, hourly outdoor concentrations for PM_2.5_, EC, NO_x_, CO were modeled at all Census block centroids in three counties (Durham, Orange, Wake) in central NC using a previously described urban-scale AQM that combines the Research LINE source dispersion model (R-LINE) and Space-Time Ordinary Kriging (STOK) model [17,19,20]. We conducted model simulations to estimate concentrations from on-road vehicle emissions, concentrations from background, and total ambient concentrations. The AQM concentrations at each participant’s home were obtained from the Census block concentrations corresponding to the home location. The details of the method are described elsewhere [17].

Briefly, the R-LINE model was used to model the concentrations from on-road sources. The traffic emissions from road segments were treated as line sources and calculated using a combination of road network, traffic activity, and pollutant-specific emission factors from EPA’s Mobile Vehicular Emission Simulator (version 2010b) [21,22]. The emission factors are categorized by road type, vehicle type, vehicle speed, and ambient temperature, which are required to calculate the actual emission from a specific roadway. These data were collected from multiple sources including the Federal Highway Administration’s road network, National Weather Service’s hourly meteorological observations, and EPA’s National Emissions Inventories [23].

The STOK model was used to model the background concentrations from all sources except for on-road vehicle emission. Following the method developed by Arunachalam et al. [20], STOK was used to interpolate monitoring data from EPA’s Air Quality System to Census block centroids [24]. This technique assumes that the concentration value at each estimation point is a linear combination of nearby observational data. The linear combination, also known as kriging weight, is determined by minimizing the estimation variance while satisfying the unbiased constraint. The STOK technique is implemented with Bayesian Maximization Entropy library [25]. The background concentration was added to the modeled on-road contribution to determine the total ambient concentration.

#### 2.2.2 Home indoor concentrations (Tier 2)

For Tier 2, hourly home indoor concentrations (*C*_in_home_) for ambient PM_2.5_, EC, NO_x_, CO were determined from home outdoor concentrations (*C*_out_home_; Tier 1) with a dynamic mass-balance infiltration model described by

(1) $$dC_{\mathrm{in\_home}}/\mathrm{dt}=AER\;P\;C_{\mathrm{out\_home}}–\left(AER+k_{r}\right)C_{\mathrm{out\_home}}$$

where *AER* is the hourly air exchange rate (h^−1^), *P* is the penetration coefficient (dimensionless), *k*_r_ is the indoor removal rate (h^−1^) [11,14]. For PM_2.5_, *P* and *k*_r_ were previously estimated from homes in the same region of NC as CADEE (*P* = 0.84, *k*_r_ = 0.21 h^−1^) [11,12]. For EC, NO_x_, CO, *P* and *k*_r_ were obtained from literature-reported values (*P* = 0.98, 1.00, 1.00; *k*_r_ = 0.29, 0.5, 0 h^−1^; respectively) [26–28]. The 24-h average *C*_in_home_ was calculated by averaging the hourly *C*_in_home_ across 24 hours.

The hourly *AER* for each participant’s home was determined from questionnaires and weather using the extended Lawrence Berkeley Laboratory model (LBLX) [11,12,14,15,17]. The AER model is mechanistic by accounting for the physical driving forces of the airflows (i.e., pressure difference across building envelope from indoor-outdoor temperature differences, called the stack effect, and from wind). The LBLX model includes leakage airflow through unintentional openings in a building envelope (e.g., cracks around windows, doors), natural ventilation through controlled openings in the building envelope (e.g., open windows, doors), and mechanical ventilation from window fans.

The LBLX model was previously described and evaluated for homes in the same region of NC as CADEE [11,12,14]]. Briefly, the leakage airflow is defined as

(2) $$Q_{\text{leak}}=A_{\text{leak}}{\left(k_{s}\left|T_{\text{in}}-T_{\text{out}}\right|+k_{w}U^{2}\right)}^{0.5}$$

where *A*_leak_ is the effective air leakage area, *k*_s_ is the stack coefficient, *k*_w_ is the wind coefficient, *T*_in_ and *T*_out_ are the average indoor and outdoor temperatures, respectively, and U is the average wind speed (see Supplementary Materials).

The LBLX model accounts for natural ventilation airflow on days with open windows or doors, and mechanical ventilation airflow on days with window fans operating [11–15,29]. The days with open windows or doors, and windows fans operating were determined from the questionnaires collected on the days with clinical measurements. If a participant reported open windows, doors; or use of window fans, we assumed open windows, doors; or window fans; respectively for the five days before questionnaires were collected (lag days for the subsequent health outcome analysis). The total airflow from leakage, natural ventilation, and mechanical ventilation is defined as

(3) $$Q_{\text{total}}={\left({Q^{2}}_{\text{mech}}+{Q^{2}}_{\text{leak}}+{Q^{2}}_{\text{nat}}\right)}^{0.5}$$

where *Q*_nat_ is the natural ventilation airflow through open windows or doors, *Q*_mech_ is the mechanical ventilation airflow through window fans (see Supplementary Materials). The AER is calculated as *Q*_total_ divided by building volume *V*.

#### 2.2.3 Personal outdoor concentrations (Tier 3)

For Tier 3, personal outdoor concentrations (*C*_out_personal_) at each 5-sec interval for ambient PM_2.5_, EC, NO_x_, CO were determined using a GPS-based outdoor concentration tracker (OCTrac) method. The OCTrac integrates the urban-scale AQM data with personal GPS data. The *C*_out_personal_ were determined by temporally and spatially matching the GPS data with the fine-scale outdoor concentrations. Each 5-sec GPS sample was time-matched to the corresponding 1-h outdoor concentration map of the three NC counties. Then, the outdoor concentration for each GPS geolocation (latitude, longitude) was obtained from the closest Census block centroid. OCTrac accounts for missing GPS data (e.g., when entering steel-framed buildings) by using geolocation of previous GPS sample. For the five days before GPS data were collected (lag days), the participant’s geolocations was set to the same locations as the day with GPS data. For lag days on weekends, we replaced any GPS samples obtained on weekdays at their work geolocation with their home geolocation. The 24-h average *C*_out_personal_ was calculated by averaging the 5-sec *C*_out_personal_ across 24 hours.

#### 2.2.4 Exposures (Tier 4)

For Tier 4, we determined exposures (*E*) at each 5-sec interval for ambient PM_2.5_, EC, NO_x_, CO as defined by

(4) $$E=ME_{{\text{in}}_{-}\text{home}}C_{\text{in\_home}}+\left[\left(ME_{\text{in\_work}}+ME_{\text{in\_other}}\right)F_{\text{inf\_other\_bldg}}+ME_{\text{in\_vehicle}}F_{\text{inf\_vehicle}}+ME_{\text{out}}\right]\;C_{\mathrm{out}\_\mathrm{personal}}$$

where *F*_inf_other_bldg_ and *F*_inf_vehicle_ are the infiltration factors (dimensionless) for buildings other than homes and for vehicles, respectively. For PM_2.5_, EC, NO_x_, CO, we set *F*_inf_other_bldg_ and *F*_inf_vehicle_ to literature-reported values (*F*_inf_other_bldg_ = 0.64, 0.59, 1.00, 1.00; *F*_inf_vehicle_ = 0.44, 0.44, 0.80, 1.00; respectively) [28,30,31]. The *ME*_in_home_, *ME*_in_work_, *ME*_in_other_, *ME*_in_vehicle_, *ME*_out_ are binary indicator variables (dimensionless) for the participant’s microenvironment (ME) at each 5-sec interval, which correspond to the five ME (indoors at home, work, other; inside vehicles; and outdoors; respectively). To simplify Equation 4, we combined the three ME associated with outdoors (outdoors at home, work, other) into one ME (outdoors). For the five days before GPS data were collected (lag days), the participant’s ME was set to the same values as the day with GPS data. For lag days on weekends, we replaced any *ME*_in_work_ = 1 with *ME*_in_home_ = 1. The 24-h average *E* were calculated by averaging the 5-sec *E* across 24 hours.

The participant’s ME at each 5-sec interval was determined using the MicroTrac model, which was previously described and evaluated for participants living in the same region of NC as CADEE [18]. Briefly, MicroTrac is a classification model that uses GPS data and geocoded building boundaries to determine the participant’s ME. The MicroTrac determines which one of seven ME (indoors and outdoors at home, work, other; inside vehicles) corresponds to the participant’s location at each 5-sec GPS sampling interval. In a previous study, MicroTrac estimates were compared with 24-h diary data from nine participants in central NC. MicroTrac correctly classified the ME for 99.5% of the daily time spent by the participants [18].

#### 2.2.5 Inhaled Doses (Tier 5)

For Tier 5, we determined inhaled doses at each 5-sec interval for ambient PM_2.5_, EC, NO_x_, CO as defined by

(5) $$D_{i}=E_{i}MVAT/BSA$$

where *D*_i_ (μg/m^2^ body surface area) is the inhaled dose (mass; μg) normalized by the participant’s body surface area (m^2^) in ME *i* where *i* = 1, 2, 3, 4, 5, 6, 7 corresponding to indoors at home, work, other; inside vehicles; outdoors at home, work, other; respectively. The *E*_i_ is the 5-sec exposure (μg/m^3^) from each ME *i*, *MV* is the 5-sec inhaled ventilation rate (m^3^/min), *AT* is the timestep (min) that is set to 0.083 min (5 sec), and *BSA* is the participant’s body surface area (m^2^). The 24-h accumulated dose in each ME was calculated by adding the 5-sec doses across 24 hours. The total 24-h accumulated dose was calculated by adding the 24-h accumulated dose in each ME.

The 5-sec exposures from each ME are defined as

(6) $$E_{1}=ME_{{\text{in}}_{-}\text{home}}C_{\text{in\_home}}$$

(7) $$E_{2}=ME_{\text{in\_work}}F_{\text{inf\_other\_bldg}}C_{\mathrm{out}\_\mathrm{pers}}$$

(8) $$E_{3}=ME_{{\text{in}}_{-}\text{other}}F_{{\text{inf}}_{-}{\text{other}}_{-}\text{blag}}C_{\mathrm{out}\_\mathrm{pers}}$$

(9) $$E_{4}=ME_{\text{in\_vehicle}}F_{\text{inf\_vehicle}}C_{\mathrm{out}\_\mathrm{pers}}$$

(10) $$E_{5}=ME_{{\text{out}}_{-}\text{home}}C_{{\text{out}}_{-}\text{pers}}$$

(11) $$E_{6}=ME_{{\text{out}}_{-}\text{work}}C_{\mathrm{out}\_\mathrm{pers}}$$

(12) $$E_{7}=ME_{{\text{out}}_{-}\text{other}}C_{\mathrm{out}\_p\mathrm{ers}}$$

where *E*_i_ is the exposure from each ME *i* where *i* = 1, 2, 3, 4, 5, 6, 7 corresponding to indoors at home, work, other; inside vehicles; outdoors at home, work, other; respectively.

To determine the participant’s *MV* at each 5-sec interval, we developed the VTrac model. First, VTrac uses accelerometer data and the GPS-based MicroTrac model, as described above, to determine which one out of four physical activity intensity levels (PAL; sedentary, light, moderate, vigorous) corresponds to the participant’s activity level. At each 5-sec interval, we added the 1-sec accelerometer activity counts across the past 60 sec (cpm; counts/min), and set the corresponding PAL based on literature-reported thresholds (sedentary: cpm<100, light: 100≤cpm<1535, moderate: 1535≤ cpm<3962, vigorous: cpm≥3962) [32]. These reported PAL thresholds were determined specifically for the Actical accelerometer used in CADEE, and were based on metabolic equivalent (METS) thresholds (sedentary: METS<2.0, light: 2.0≤METS<3.0, moderate: 3.0≤METS<6.0, vigorous: METS≥6.0). For the five days before accelerometer data were collected (lag days), the participant’s activity counts were set to the same values as the day with accelerometer data.

To account for possible misclassifications when the participant is inside vehicles, we used the ME determined from the GPS-based MicroTrac. We set the PAL to sedentary when the time-matched ME is classified as inside vehicles, since the accelerometer may detect motion from the vehicles even though the participant is sitting (i.e., sedentary) inside a vehicle.

The VTrac model then determines age- and sex-specific *MV* for each PAL based on literature-reported normalized minute ventilation (*NMV*) (L/min/kg body weight) [33]. The *NMV* were determined from oxygen consumption rates and basal metabolic rates based on data from the National Health and Nutrition Examination Survey and EPA’s Consolidated Human Activity Database. The *NMV* were reported for: (1) each of the four PAL based on METS thresholds (sedentary: METS≤1.5, light: 1.5<METS≤3.0, moderate: 3.0<METS≤6.0, vigorous: METS>6.0), (2) 14 separate age categories, (3) both males and females. For CADEE, we used the reported median *NMV* for each PAL based on the participant’s age and sex. The *MV* is calculated as *NMV* multiplied by the participant’s body weight (kg).

The BSA is defined as

(13) $$\text{BSA}\;=\;0.007184\;BH^{0.725}BW^{0.425}$$

where *BH* is body height (cm) and *BW* is body weight (kg) [34].

## 3. Results

To apply the fine-scale exposure model for CADEE, we modeled five tiers of daily exposure metrics for all 15 study participants and their homes. Modeled concentrations of PM_2.5_, EC, NO_x_, and CO for daily 24-h averages (8am – 8am) are provided, which are time-matched to the daily health measurements for a future epidemiological analysis. We modeled a total of 720 participant-days.

**Figure 2.**
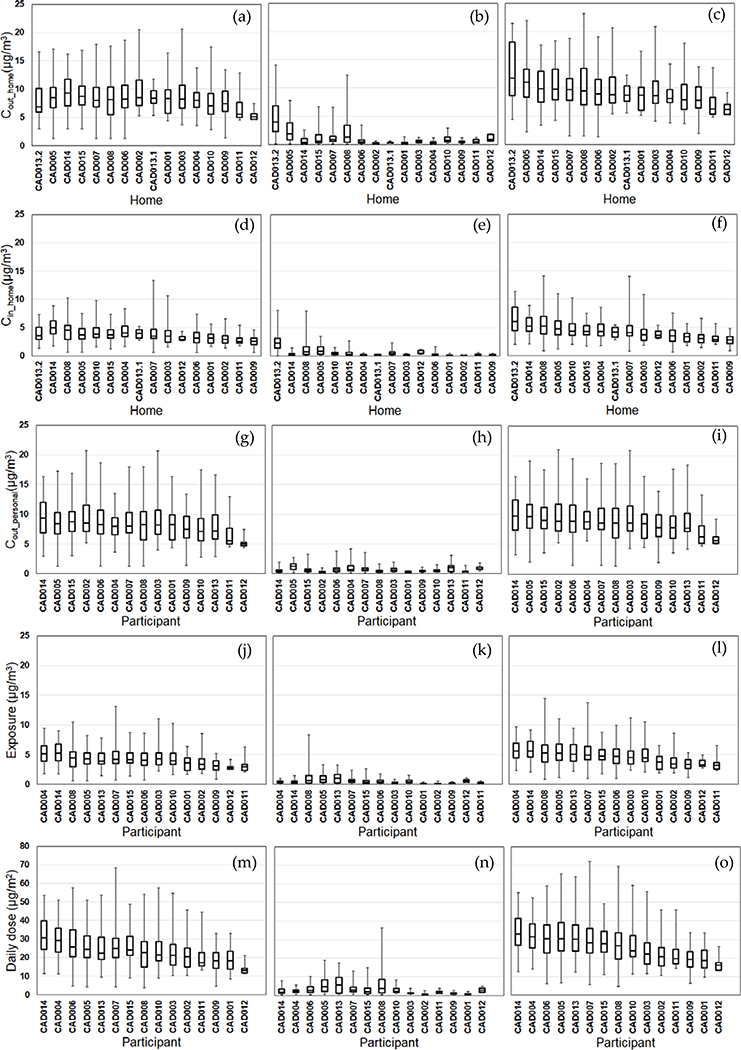
Modeled PM_2.5_ exposure metrics for Tier 1 (outdoor home concentrations; a-c), Tier 2 (indoor home concentrations; d-f), Tier 3 (personal outdoor concentrations; g-i), Tier 4 (exposures; j-l), Tier 5 (inhaled dose; m-o) from background (left), on-road vehicle emissions (middle), and total PM_2.5_ (right). Results (24-h average, 8am-8am) are sorted by total PM_2.5_ median values from highest to lowest. Shown are medians with 25^th^ and 75^th^ percentiles, and whiskers for minimum and maximum values.

**Figure 3.**
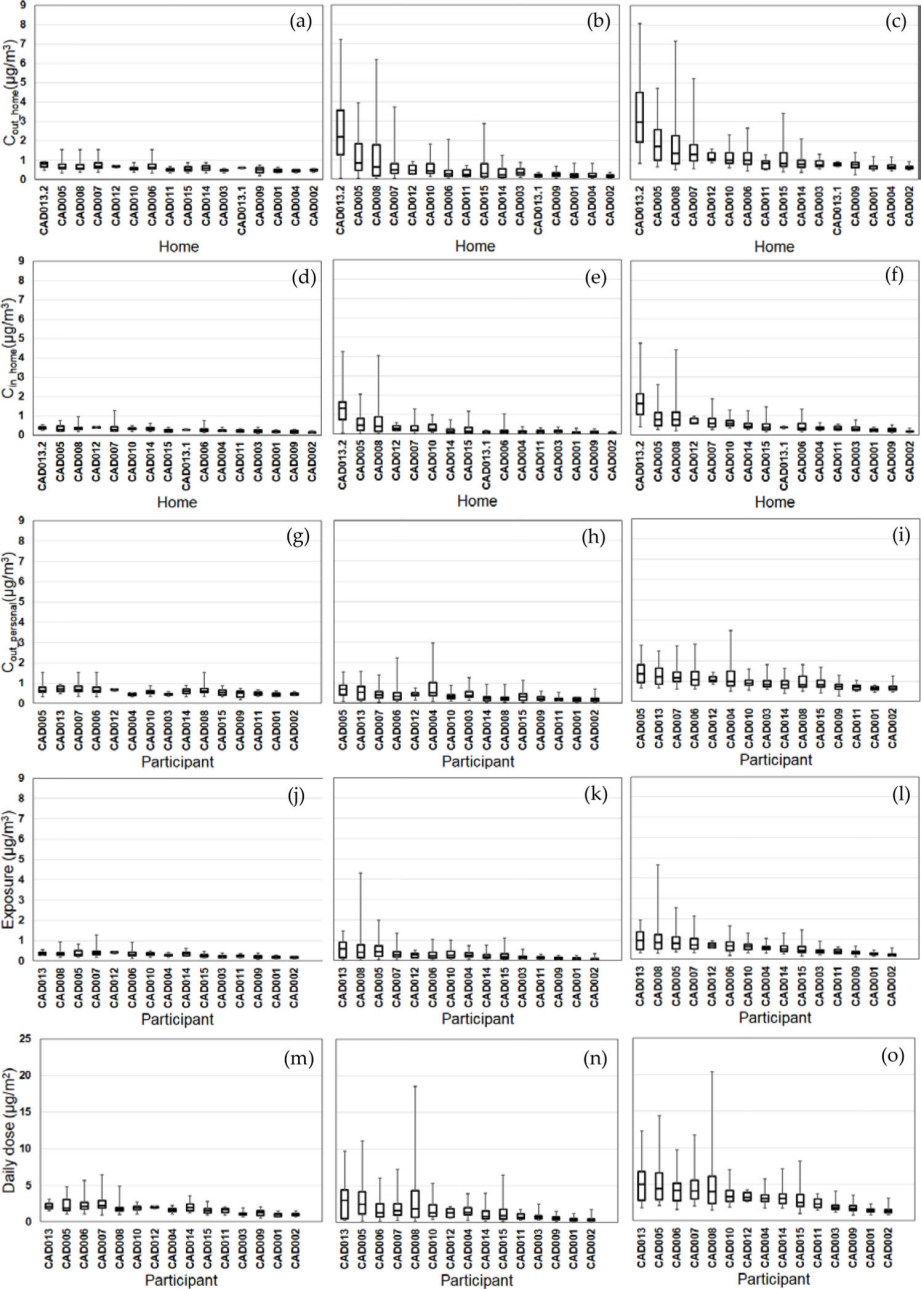
Modeled EC exposure metrics for Tier 1 (outdoor home concentrations; a-c), Tier 2 (indoor home concentrations; d-f), Tier 3 (personal outdoor concentrations; g-i), Tier 4 (exposures; j-l), Tier 5 (inhaled dose; m-o) from background (left), on-road vehicle emissions (middle), and total EC (right). Results (24-h average, 8am-8am) are sorted by total EC median values from highest to lowest. Shown are medians with 25^th^ and 75^th^ percentiles, and whiskers for minimum and maximum values.

**Figure 4.**
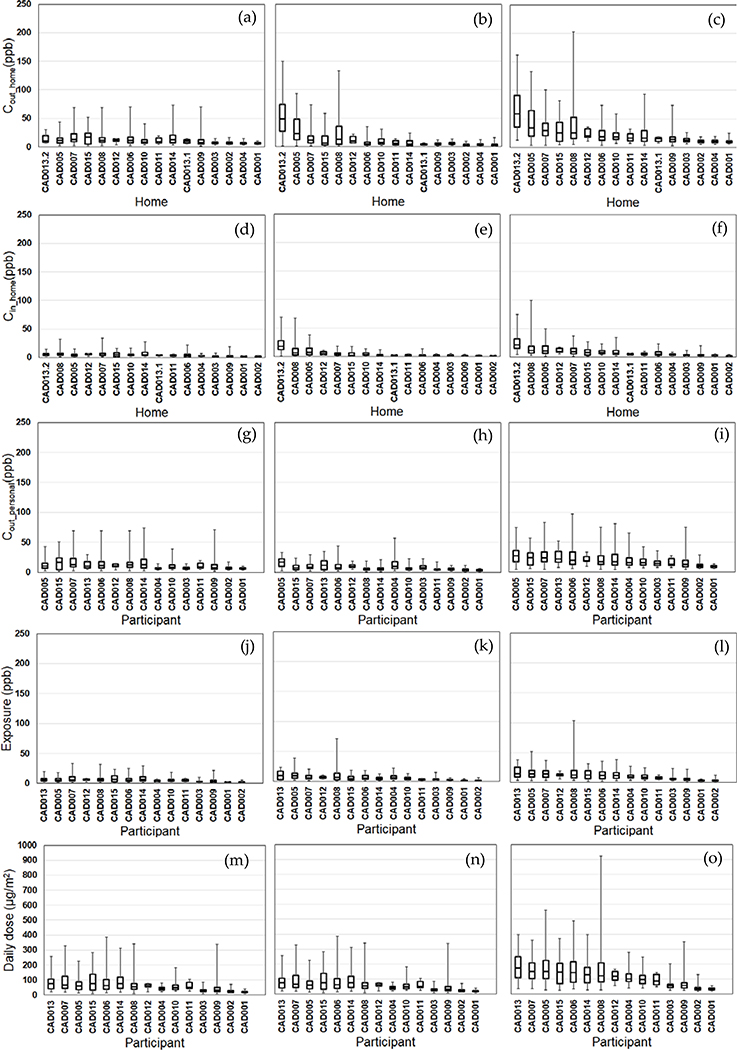
Modeled NO_x_ exposure metrics for Tier 1 (outdoor home concentrations; a-c), Tier 2 (indoor home concentrations; d-f), Tier 3 (personal outdoor concentrations; g-i), Tier 4 (exposures; j-l), Tier 5 (inhaled dose; m-o) from background (left), on-road vehicle emissions (middle), and total NO_x_ (right). Results (24-h average, 8am-8am) are sorted by total NO_x_ median values from highest to lowest. Shown are medians with 25^th^ and 75^th^ percentiles, and whiskers for minimum and maximum values.

**Figure 5.**
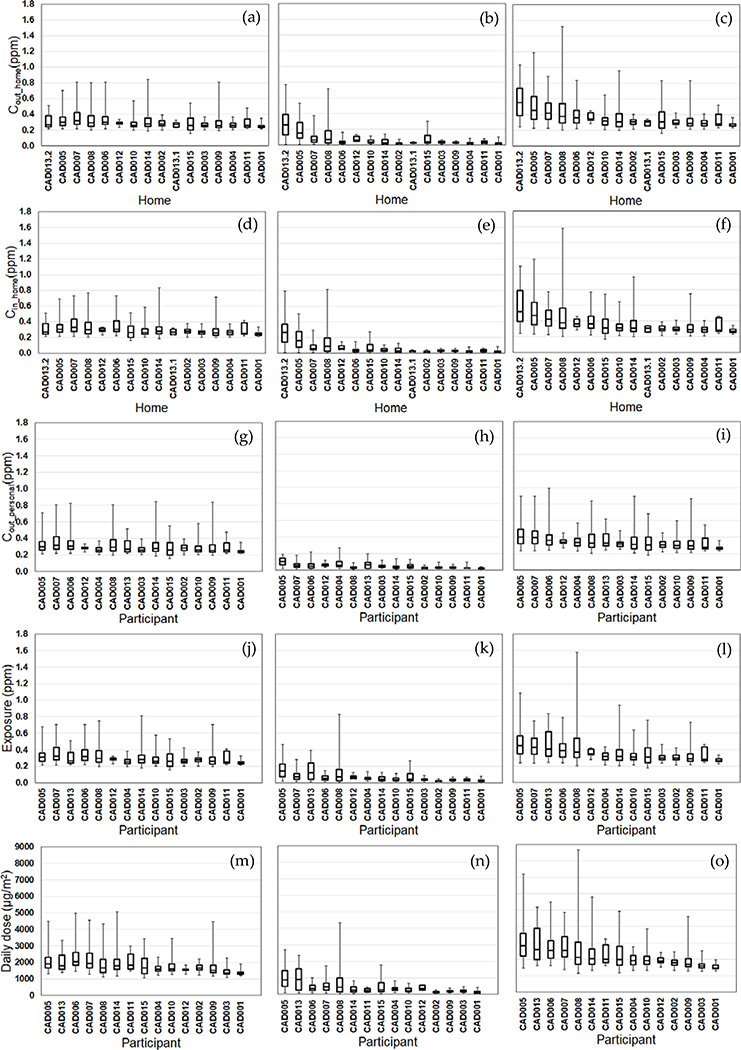
Modeled CO exposure metrics for Tier 1 (outdoor home concentrations; a-c), Tier 2 (indoor home concentrations; d-f), Tier 3 (personal outdoor concentrations; g-i), Tier 4 (exposures; j-l), Tier 5 (inhaled dose; m-o) from background (left), on-road vehicle emissions (middle), and total CO (right). Results (24-h average, 8am-8am) are sorted by total CO median values from highest to lowest. Shown are medians with 25^th^ and 75^th^ percentiles, and whiskers for minimum and maximum values.

We compared the daily variability of the modeled exposure metrics for individual homes (Tiers 1–2) and participants (Tiers 3–5) (Figures 2–5). For Tier 1, the temporal variability (within homes) and home-to-home variability of *C*_out_home_ was substantial for all four pollutants due to daily variations. Also, the on-road contribution to total *C*_out_home_ was larger than the background contribution for EC and NO_x_, and smaller for PM_2.5_ and CO due to substantial on-road emissions and near-road spatial gradients of EC and NO_x_.

For Tier 2, *C*_in_home_ was substantially lower than *C*_out_home_ for PM_2.5_, EC, and NO_x_, but the same for CO due to the home indoor attenuation of ambient PM_2.5_, EC, and NO_x_, but no indoor attenuation of CO. In the plots of the homes ranked by median *C*_in_home_ and *C*_out_home_, the order of the homes for *C*_in_home_ was different than *C*_out_home_ for PM_2.5_, EC, and NO_x_ due to the temporal and home-to-home variability of the residential AER from indoor-outdoor temperature differences, wind speed, and building operating conditions (e.g., open windows). The home-to-home variability was also due to building leakage area differences.

For Tier 3, *C*_out_personal_ was substantially different than *C*_out_home_ for EC, NO_x_, and CO, but similar for PM_2.5_ is due to the larger spatial variability of EC, NO_x_, and CO as compared to PM_2.5_. Also, the participant-to-participant variability between *C*_out_personal_ and *C*_out_home_ is due to time-of-day and duration at geolocations other than home.

For Tier 4, *E* was substantially lower than *C*_out_home_ for PM_2.5_, EC, and NO_x_, but the same for CO due to the indoor attenuation of ambient PM_2.5_, EC, and NO_x_, but no indoor attenuation of CO. In the plots of the participants ranked by median *E* and *C*_out_home_, the order of the participants for *E* was different than *C*_out_home_ for PM_2.5_, EC, and NO_x_ due to the temporal and participant-to-participant variability of time spent outdoors and within indoor microenvironments other than home and with different infiltration factors.

For Tier 5, the background contribution to total *D* was larger than the on-road contribution for PM_2.5_ and CO, smaller for EC, and similar for NOx. Also, the participants with high, moderate, and low median doses tended to be similar participants for EC, CO, and NO_x_, but not for PM_2.5_.

**Figure 6.**
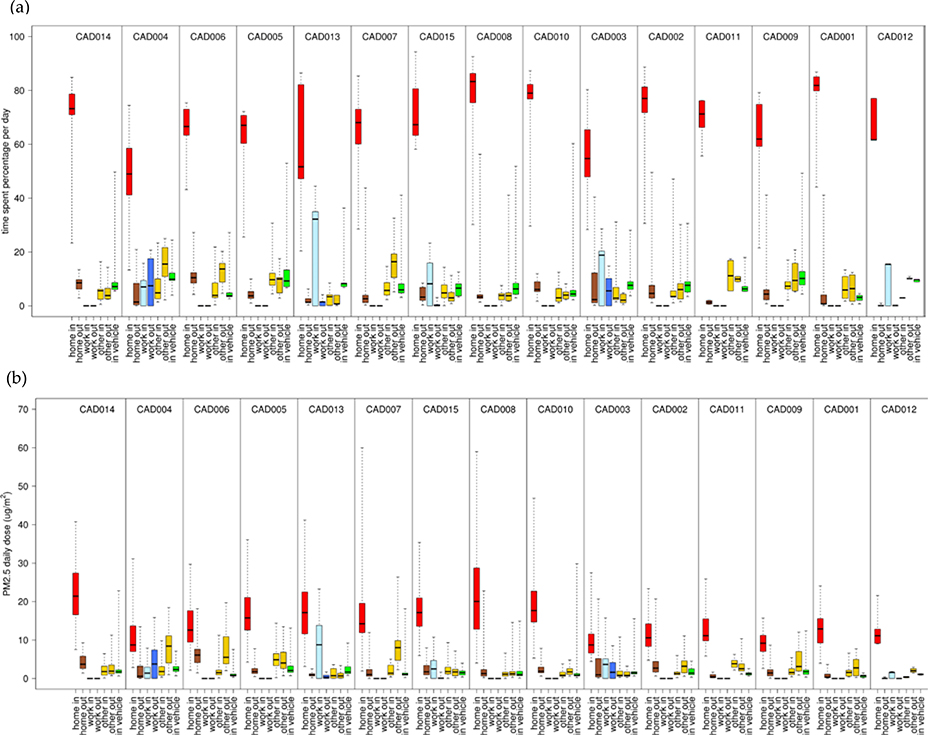
Percentage of daily time spent (a) and inhaled dose for total PM_2.5_ (b) for each microenvironment (indoors and outdoors at home, work, and other; inside vehicle) and each participant. Results (24-h average, 8am-8am) are sorted by median values of the total dose from highest to lowest. Shown are medians with 25^th^ and 75^th^ percentiles, and whiskers for minimum and maximum values.

We compared the variability of daily *D* and time spent in each ME (Figure 6, Figures S1–S4). For all participants, the highest median dose and greatest time spent was indoors at home. For the other six ME, the ME with greater time spent usually corresponded to higher median dose for most participants. For CO, this was always the case. For PM_2.5_, EC, and NO_x_ and for a few participants, the three indoor ME (work, other, in-vehicles) with greater time spent corresponded to lower median doses as compared to the three outdoor ME (home, work, other). This is likely due to the indoor and in-vehicle attenuation of ambient PM_2.5_., EC, and NO_x_, whereas ambient CO has no indoor or in-vehicle attenuation [26–28]. Also, the daily physical activity levels had substantial temporal and participant-to-participant variability for daily time-spent performing at low intensity levels (e.g., walking) with an overall range of 20–390 min/day (Figure S5).

## 4. Discussion

Our goal was to determine daily ambient PM_2.5_, EC, NO_x_, and CO exposure metrics for each CADEE participant in support of improving health effect estimation for future epidemiological analysis. Using a fine-scale exposure model, we performed an individual-level exposure assessment in CADEE that accounts for daily variations in ambient PM_2.5_, EC, NO_x_, and CO exposures separated by background, on-road vehicle emissions, and total concentrations based on an urban-scale AQM, a mechanistic house-specific AER model linked to a mass-balance infiltration model, infiltration factors for nonresidential buildings and vehicles, GPS-based microenvironment model, and accelerometer-based inhaled ventilation model. The impact of applying our fine-scale exposure model for an epidemiological study to improve health effect estimation will depend on various factors such as the health study design and the true exposure distributions [35,36]. We predicted multiple tiers of exposure metrics with different levels of complexity and uncertainty, which will be used in the epidemiological analysis to help determine the benefit of more sophisticated exposure metrics.

There are several benefits of using EMI for panel studies, such as CADEE, with individual-level health outcomes. First, spatio-temporal exposure models are needed that account for time-location variability of individuals that transition between microenvironments with different ambient pollutant concentrations. The National Research Council report: “Exposure Science in the 21^st^ Century” highlighted the need for spatio-temporal exposure models that use input data for time-locations, housing characteristics, and ambient concentrations [7]. Second, population-level exposure models (e.g., SHEDS, APEX) predict exposures for demographic groups using population-level inputs from other studies, such as the U.S. Census [30,37,38]; whereas EMI predicts exposures for specific individuals in an epidemiological study using individual-level input data (e.g., questionnaires, time-location diaries) from each study participant. Thus, population exposure models are appropriate for studies with number of health outcomes across a region. The EMI is appropriate for panel studies, including studies that use personalized exposure, and genetic and cellular data to determine the role of individual susceptibility and effect modifiers on adverse responses to the four air pollutants [39]. The need for exposure models that are specific to susceptible individuals, such as people with cardiovascular and pulmonary disease, was highlighted in the National Research Council report on exposure science [7].

For exposure models, there are two types of measurement errors that can impact health effect estimates [6,35]. Berkson-like errors are from using a model that is missing some sources of variation or exposure factors. Classical-like errors are from uncertainty in the estimated model parameters. These errors can bias health effect estimates and alter confidence levels. Using our exposure modeling approach can minimize both types of errors. The urban-scale AQM can reduce Berkson-like error by accounting for spatio-temporal variability of outdoor concentrations. Our mechanistic AER model can reduce Berkson-like error by accounting for the home-to-home variations due to building characteristics and operation (e.g., window opening) and the temporal variations due to stack and wind effects [11,14]. The GPS-based MicroTrac model can also reduce Berkson-like error by accounting for the daily participant-to-participant variations in the time spent in various microenvironments with different infiltration factors [18]. Classical-like error can be reduced with our previous PM_2.5_ model calibration and evaluation to improve the estimated parameters of the mass balance residential infiltration model [11,12,14], and our previous evaluation of the MicroTrac model [18].

Variability in home infiltration of ambient air pollutants and subject time-location patterns that contribute to exposure variability can impact epidemiological studies [7]. Sarnat et al. accounted for the spatio-temporal variability of residential AER in Atlanta, and found associations for the interaction between daily zip code-level AER and outdoor PM_2.5_, NOx, and CO concentrations on asthma emergency department visits [40]. Kaufman et al. accounted for temporal and house-to-house variability of PM_2.5_ infiltration and subject-specific time spent indoors for >6,000 participants, and found significant associations between individual-level ambient PM_2.5_ exposures and coronary artery calcification [41]. Koenig et al. also accounted for temporal and house-to-house variability of PM_2.5_ infiltration and daily time spent indoors for children with asthma, and found ambient PM_2.5_ exposures were significantly associated with increases in exhaled nitric oxide [42]. These studies demonstrate the importance of accounting for individual-level exposure variability in epidemiological studies.

One limitation of this study is the exposure metrics do not include non-ambient air pollutants. Wilson et al. showed the importance of separating ambient and non-ambient pollutant exposures since the EPA regulates only ambient pollutants, and pollutants from ambient and non-ambient sources have different chemical properties (particulate matter only) and temporal patterns, which can induce different health effects [43]. When we apply these modeled exposure metrics for epidemiological analysis, we plan to separately examine factors associated with non-ambient sources (e.g., gas stoves, environmental tobacco smoke) as categorical variables in the epidemiological models, which can remove potential uncertainties in modeled exposures that include indoor sources.

Another potential limitation of this study is the exposure model uses outdoor air pollutant concentrations from a sophisticated urban-scale air quality model that requires substantial expertise and resources. For air pollutants that are spatially homogeneous (e.g., PM_2.5_), using fixed-site monitor measurements as inputs for the exposure model may be sufficient in certain geographical regions, except near large sources that can produce substantial spatial variations. In a previous study in central NC, we found no substantial difference between daily ambient PM_2.5_ exposures determined from a fixed-site PM_2.5_ monitors and those predicted from PM_2.5_ monitors outside each participant’s home [11]. For other air pollutants that can have substantial local spatial and temporal variations from nearby sources such as traffic (e.g. EC, NO_x_, CO), a fine-scale air quality model can account for this spatio-temporal variability. To facilitate and expand the use of exposure models for epidemiological studies, we developed a smartphone-based exposure model, called TracMyAir that determines individual-level exposure metrics for ambient PM_2.5_ and ozone [13]. The TracMyAir uses the smartphone’s geolocations to obtain real-time input data from the nearest outdoor air monitors. We plan to expand TracMyAir to automatically input data from urban-scale air quality models for other air pollutants with spatio-temporal variability.

Another potential limitation is that for the five lags days before GPS data were collected, the participant’s ME was set to the same values as the day with GPS data. For lag days on weekdays, we expect only small changes in daily time spent in different ME since the GPS data was also collected on weekdays. For lag days on weekends, we replaced any time spent indoors at work with time spent indoors at home. To further examine how changes in the time spent in various ME affect the resulting exposure, we performed a sensitivity analysis. The details of the method are described in Supplementary Materials. The sensitivity analysis showed that large changes in the time spent in ME with substantially different infiltrations (e.g., indoors versus outdoors) can produce large changes in the exposures for PM_2.5_, EC, NO_x_, but have little or no effect on exposures to CO since infiltrations are similar for all ME. In this study, we expect small difference in the time spent in ME on the lag days versus the day with GPS data. To reduce this potential exposure uncertainty, we developed a smartphone application for our exposure model called TracMyAir that will be used in future epidemiological studies to facilitate the collection of daily long-term time-location data [13].

## 5. Conclusions

This study demonstrates the ability of applying a fine-scale exposure model to determine five tiers of individual-level exposure metrics for the homes and participants in an epidemiological study. To improve exposure assessments in CADEE, EMI accounts for (1) hourly Census block outdoor concentrations for four ambient pollutants, (2) hourly house-specific infiltrations, (3) continuous (5-sec) participant-specific time locations for seven ME (indoors and outdoors at home, work, other; inside vehicles), and (4) continuous participant-specific inhaled ventilations. This capability can help improve exposure assessments for epidemiological studies, such as CADEE, in support of human health risk assessments.

## Supplementary Material

supplemental file
