# Supplementary material for: Fine-Scale Modeling of Individual Exposures to Ambient PM2.5, EC, NOx, CO for the Coronary Artery Disease and Environmental Exposure (CADEE) Study
Source: Atmosphere (Basel). Author manuscript; Available in PMC 2020 May 27. (PMC7252567; doi:10.3390/atmos11010065)
Supplement: supplemental file [file NIHMS1551998-supplement-supplemental_file.pdf]

atmosphere

Supplementary for

Fine-Scale Modeling of Individual Exposures to Ambient $PM_{2.5}$, EC, NO_x, and CO for the Coronary Artery Disease and Environmental Exposure (CADEE) Study

Michael Breen [1,*], Shih Ying Chang [2], Miyuki Breen [3], Yadong Xu [4], Vlad Isakov [5], Saravanan Arunachalam [2], Martha Sue Carraway [6] and Robert Devlin [7]

[1] Center for Public Health and Environmental Assessment, U.S. Environmental Protection Agency, Research Triangle Park, NC 27711, USA
[2] Institute for the Environment, University of North Carolina at Chapel Hill, Chapel Hill, NC 27517, USA; cchang@sonomatech.com (S.Y.C.); sarav@email.unc.edu (S.A.)
[3] Oak Ridge Institute for Science and Education (ORISE), U.S. Environmental Protection Agency, Chapel Hill, NC 27514, USA; breen.miyuki@epa.gov
[4] Oak Ridge Associated Universities (ORAU), U.S. Environmental Protection Agency, Research Triangle Park, NC 27711, USA; xu.yadong@epa.gov
[5] Center for Measurements and Modeling, U.S. Environmental Protection Agency, Research Triangle Park, NC 27711, USA; isakov.vlad@epa.gov
[6] Department of Medicine, Pulmonary and Critical Care Medicine, Durham VA Medical Center, Durham, NC 27705 USA; martha.carraway@duke.edu
[7] Center for Public Health and Environmental Assessment, U.S. Environmental Protection Agency, Chapel Hill, NC 27514, USA; devlin.robert@epa.gov
* Correspondence: breen.michael@epa.gov; Tel.: +1-919-541-9409

Received: 30 November 2019; Accepted: 31 December 2019; Published: date

Air Exchange Rate Model

The AER has two parameters (k_s and k_w) and five inputs (A_{leak}, T_{in}, T_{out}, U, and V). Parameters k_s and k_w were set to literature-reported values based on house-specific information on house height (number of stories) and local wind sheltering (Supplementary Material, Tables S1-S3). Using home addresses, the number of stories and local wind sheltering were determined from satellite and street-level images in Google Earth (version 7.1.7.2606; Google, Mountain View, CA, USA). We used house numbers visible in street-level images to verify the participant homes. The number of stories was verified from online county and real estate databases of property records (Zillow, Seattle, WA, USA; Trulia, San Francisco, CA, USA). To determine V, we multiplied floor area by a ceiling height of 2.44 m (8 ft). The floor area was obtained from the online county and real estate databases.

We determined T_{out} and U (10 m elevation) from hourly measurements at Raleigh Durham Airport in Morrisville, NC, USA. We calculated 24 h average T_{out} and U time-matched to the 24 h average $PM_{2.5}$ measurements. We determined T_{in} from daily values from the daily participant questionnaires.

We estimate A_{leak} with a literature-reported leakage area model [1,2]. The A_{leak} is calculated as

$$A_{leak} = \frac{NL}{NF} \text{ [S1]} \tag{1}$$

where NL is the normalized leakage and NF is the normalization factor. The NL is predicted from year of construction Y_{built} and floor area A_{floor} as described by

$$NL=\exp(\beta_0+\beta_1 Y_{built}+\beta_2 A_{floor})\ [S2] \tag{2}$$

where β_0, β_1, and β_2 are regression parameters. The NF is defined as

$$NF = \frac{1000}{A_{floor}}\left(\frac{H}{2.5}\right)^{0.3}\ [S3] \tag{3}$$

where H is the building height. We set H to the number of stories multiplied by a story height of 2.5 m and adding a roof height of 0.5 m (Breen et al., 2010). The A_{floor} and Y_{built} were obtained from online county and real estate databases of property records as described above.

The parameters β_0, β_1, and β_2 were estimated by Chan et al. (2005) [2] for low-income homes (β_0=11.1, β_1=-5.37x10^{-3}, and β_2=-4.18x$10^{-3}$$m^{-2}$) and conventional homes (β_0=20.7, β_1=-1.07x10^{-2}, and β_2=-2.20x$10^{-3}$$m^{-2}$). Low-income homes were defined as residences with household incomes below 125% of the poverty guideline. In DEPS, the individual household incomes were not collected. Using 2010 U.S. Census, we examined the median household income at the block group for each home. The household incomes were all substantially above 125% of the 2010 poverty guideline. Therefore, we used the literature-reported parameters for conventional homes in CADEE.

For the airflow from natural ventilation Q_{nat} can be calculated as:

$$Q_{nat} = \sqrt{Q_{nat,wind}^2 + Q_{nat,stack}^2}\ [S4] \tag{4}$$

where $Q_{nat,wind}$ and $Q_{nat,stack}$ are the airflows from the wind and stack effects, respectively. The $Q_{nat,wind}$ is defined as:

$$Q_{nat,wind} = C_v A_{nat} U \quad [S5] \tag{5}$$

where C_v is the effectiveness of the openings, and the A_{nat} is the area of the inlet openings. Using literature-reported values, we set C_v to 0.3 and A_{nat} to one-half of the total area of window and door openings (Breen et al. 2010). The daily participant questionnaires were used to determine number and duration that windows and doors were opened. Window and door opening areas were not collected in CADEE. For windows, we set A_{nat} to one-half of the literature-reported value of 619 cm^2, which is the median daily total window opening area for homes in the same region of central NC as DEPS (Breen et al. 2010). For doors, we set A_{nat} to one-half of 3600 cm^2. The $Q_{nat,stack}$ is defined as:

$$Q_{nat,stack} = C_D A_{nat}\sqrt{2g\Delta H_{NPL}|T_{in} - T_{out}|/max\{T_{in}, T_{out}\}}[S6] \tag{6}$$

where C_D is the discharge coefficient for the openings, g is the gravitational acceleration, ΔH_{NPL} is the height from midpoint of lower window opening to the neutral pressure level (NPL) of the building, and $max\{T_{in}, T_{out}\}$ is the maximum value between T_{in} and T_{out}. Using literature-reported values, we set C_D to 0.65, midpoint of lower window opening to 0.91 m, and NPL to one-half of H [1].

For the days with operating window fans, the airflow (Q_{total}) was calculated as follows:

$$Q_{total} = Q_{bal} + \sqrt{Q_{unbal}^2 + Q_{leak}^2 + Q_{nat}^2} \tag{7}$$

where Q_{bal} and Q_{unbal} are balanced and unbalanced flow rate respectively, Q_{leak} is the flow from leakage, and Q_{nat} is the flow from natural ventilation. The daily participant questionnaires were used to determine number and duration that window fans were operated. Since whether the window fan system is balanced (i.e. pair of intake and exhaust fan) or unbalanced (i.e. a single intake or exhaust fan) was not recorded, we assume an unbalanced system for all houses with window fan operating (Q_{bal}=0). Q_{unbal} was set at 600 ft^3/min for each window fan, which is the mid-range value for medium-size window fans (range: 300-900 ft^3/min) [3].

Sensitivity Analysis

For the sensitivity analysis of time spent in different microenvironments, we determined exposures (E) as defined by

$$E = F_{pex}\ C_{out} \tag{8}$$

where F_{pex} is the personal exposure factor (dimensionless), and C_{out} is the outdoor concentration. The F_{pex} is defined by

$$F_{pex} = f_{in\_home}\ F_{inf\_home} + (f_{in\_work} + f_{in\_other\_bldg})F_{inf\_other\_bldg} + f_{in\_vehicle}\ F_{inf\_vehicle} + f_{out}\ F_{out}$$

where f_{in_home}, f_{in_work}, $f_{in\_other\_bldg}$, $f_{in\_vehicle}$, f_{out} are the fraction of day spent in indoors at home, work, other buildings; inside vehicles; outdoors; respectively; and F_{inf_home}, $F_{inf\_other\_bldg}$, $F_{in\_vehicle}$, F_{out} are the infiltration (i.e., attenuation) factors for home, other buildings including work, vehicles, outdoors, respectively. The F_{inf_home} is defined by a steady-state mass-balance infiltration model described by

$$F_{inf\_home} = (P\ AER)/(AER + k_r) \tag{9}$$

where P is the penetration coefficient (dimensionless), AER is the air exchange rate (h^{-1}), and k_r is the indoor removal rate (h^{-1}). We set AER to the median value (0.5 h^{-1}) measured from homes in the same region of North Carolina as CADEE homes [4]. We used the same parameter as described in the main paper. For $PM_{2.5}$, P and k_r were previously estimated from homes in the same region of NC as CADEE (P = 0.84, k_r = 0.21 h^{-1}) [4,5]. For EC, NO_x, CO, P and k_r were obtained from literature-reported values (P = 0.98, 1.00, 1.00; k_r = 0.29, 0.5, 0 h^{-1}; respectively) [6–8]. This yields F_{inf_home} of 0.59, 0.62, 0.50, 1.0 for $PM_{2.5}$, EC, NO_x, CO; respectively.

For $PM_{2.5}$, EC, NO_x, CO, we set $F_{inf\_other\_bldg}$ and $F_{inf\_vehicle}$ to literature-reported values ($F_{inf\_other\_bldg}$ = 0.64, 0.59, 1.00, 1.00; $F_{inf\_vehicle}$ = 0.44, 0.44, 0.80, 1.00; respectively) [8–10]. For outdoors, F_{out} is always 1.0.

For the five different microenvironments that participants can spend their time, the infiltration factors (F_{inf_home}, $F_{inf\_other\_bldg}$, $F_{inf\_vehicle}$, F_{out}) vary by a range (max-min) and factor (max/min) of (0.44-1.00; factor of 2.3) for $PM_{2.5}$, (0.44-1.00; factor of 2.3) for EC, (0.50-1.00; factor of 2.0) for NO_x, (1.00-1.00; factor of 1.0) for CO. Therefore, changes in the time spent in ME with substantially different infiltration factors (e.g., indoors versus outdoors) can produce substantial changes in the exposures for $PM_{2.5}$, EC, NO_x., but have little or no effect on exposures to CO.

Table S1. Stack coefficient[1] k_s [(L/s)^2/(cm^4 K)].

	House Height (Stories)		
	One	Two	Three
Stack coefficient	0.000145	0.000290	0.000435

[1] ASHRAE Handbook-Fundamentals, 2009.

Table S2. Wind coefficient[1] k_w [(L/s)^2/(cm^4 (m/s)^2)].

Shelter Class	House Height (Stories)		
	One	Two	Three
1	0.000319	0.000420	0.000494
2	0.000246	0.000325	0.000382
3	0.000174	0.000231	0.000271
4	0.000104	0.000137	0.000161
5	0.000032	0.000042	0.000049

[1] ASHRAE Handbook-Fundamentals, 2009 [11].

Table S3. Local Sheltering[1].

Shelter Class	Description
1	No obstructions or local sheltering
2	Typical shelter for an isolated rural house
3	Typical shelter caused by other buildings across street from building under study
4	Typical shelter for urban buildings on larger lots where sheltering obstacles are more than one building height away
5	Typical shelter produced by buildings or other structures immediately adjacent (closer than one building height): e.g., neighboring houses on same side of street, trees, bushes, etc.

[1] ASHRAE Handbook-Fundamentals, 2009 [11].

Table S4. Male sedentary ventilation rates[2]

Ventilation Rates (L/min/kg body weight)	
1 year old	0.40
2 years old	0.34
3-5 years old	0.25
6-10 years old	0.16
11-15 years old	0.10
16-20 years old	0.08
21-30 years old	0.06
31-60 years old	0.07
61-80 years old	0.08
81 years and older	0.09

[2] Breen et al., 2019 [3].

Table S5. Male light intensity ventilation rates[2]

Ventilation Rates (L/min/kg body weight)	
1 year old	1.01
2 years old	0.83

3-5 years old	0.63
6-10 years old	0.38
11-15 years old	0.24
16-20 years old	0.18
21-30 years old	0.15
31-70 years old	0.16
71-80 years old	0.17
81 years and older	0.18

[2] Breen et al., 2019 [3].

Table S6. Male moderate intensity ventilation rates[2]

Ventilation Rates (L/min/kg body weight)	
1 year old	1.82
2 years old	1.54
3-5 years old	1.12
6-10 years old	0.71
11-15 years old	0.47
16-20 years old	0.38
21-40 years old	0.34
41-50 years old	0.35
51-60 years old	0.37
61-70 years old	0.34
71-80 years old	0.36
81 years and older	0.38

[2] Breen et al., 2019 [3].

Table S7. Male vigorous intensity ventilation rates[2]

Ventilation Rates (L/min/kg body weight)	
1 year old	3.57
2 years old	2.87
3-5 years old	2.11
6-10 years old	1.38
11-15 years old	0.91
16-20 years old	0.69
21-30 years old	0.64
31-40 years old	0.62
41-50 years old	0.63
51-60 years old	0.64
61-70 years old	0.61
71-80 years old	0.63
81 years and older	0.70

[2] Breen et al., 2019 [3].

Table S8. Female sedentary ventilation rates[2]

Ventilation Rates (L/min/kg body weight)	
1 year old	0.42
2 years old	0.35
3-5 years old	0.25
6-10 years old	0.16
11-15 years old	0.09
16-20 years old	0.07
21-50 years old	0.06
51-80 years old	0.07
81 years and older	0.08

[2] Breen et al., 2019 [3].

Table S9. Female light intensity ventilation rates[2]

Ventilation Rates (L/min/kg body weight)	
1 year old	1.04
2 years old	0.89
3-5 years old	0.60
6-10 years old	0.38
11-15 years old	0.22
16-20 years old	0.17
21-40 years old	0.15
41-60 years old	0.16
61-70 years old	0.14
71 years and older	0.16

[2] Breen et al., 2019 [3].

Table S10. Female moderate intensity ventilation rates[2]

Ventilation Rates (L/min/kg body weight)	
1 year old	1.87
2 years old	1.58
3-5 years old	1.11
6-10 years old	0.71
11-15 years old	0.43
16-20 years old	0.35
21-30 years old	0.32
31-40 years old	0.30
41-50 years old	0.32
51-60 years old	0.33
61-70 years old	0.28
71-80 years old	0.30
81 years and older	0.33

[2] Breen et al., 2019 [3].

Table S11. Female vigorous intensity ventilation rates[2]

Ventilation Rates (L/min/kg body weight)	
1 year old	3.24
2 years old	2.81
3-5 years old	1.90
6-10 years old	1.33
11-15 years old	0.85
16-20 years old	0.69
21-30 years old	0.63
31-40 years old	0.59
41-50 years old	0.64
51-60 years old	0.61
61-70 years old	0.53
71-80 years old	0.58
81 years and older	0.63

[2] Breen et al., 2019 [3].

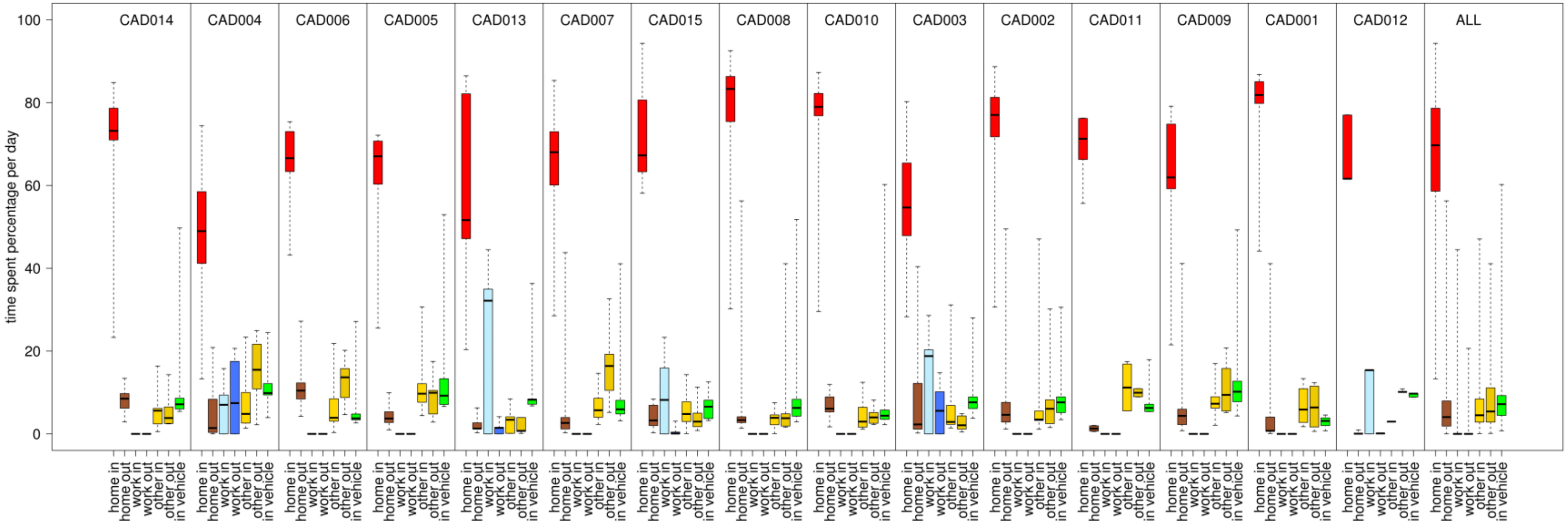

CAD014
CAD004
CAD006
CAD005
CAD013
CAD007
CAD015
CAD008
CAD010
CAD003
CAD002
CAD011
CAD009
CAD001
CAD012
ALL
time spent percentage per day
100
80
60
40
20
0
home in
home out
work in
work out
other in
other out
in vehicle

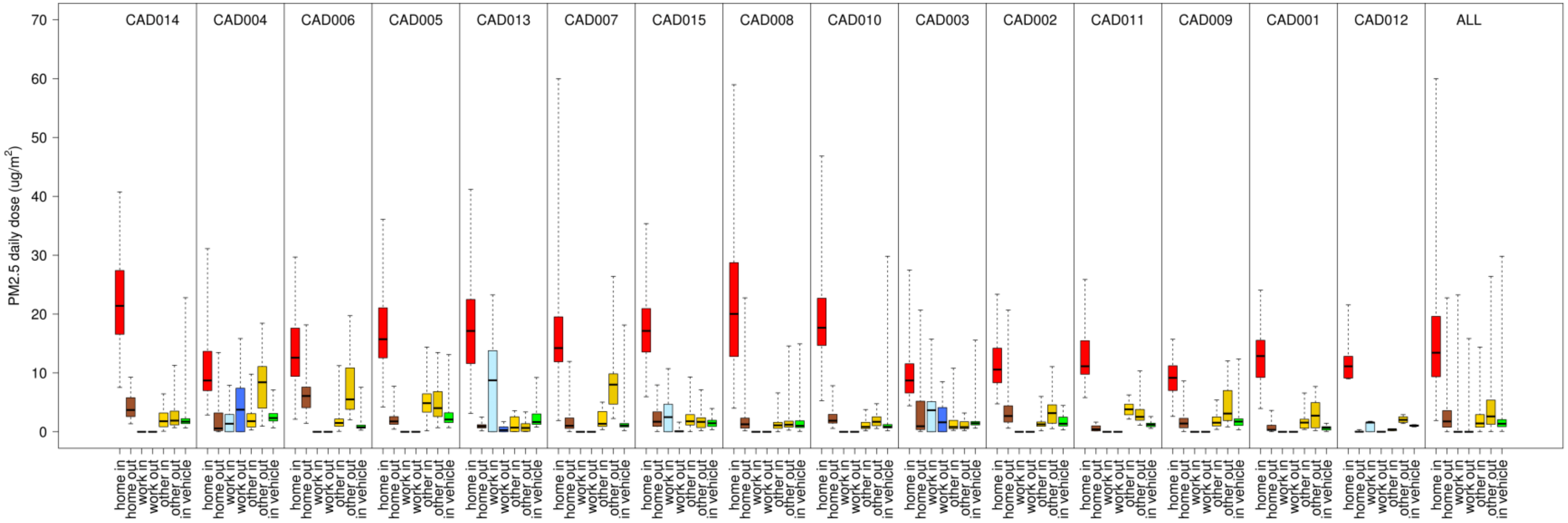

Figure 1. Percentage of time spent per day (%) (**top**) and daily dose (ug/m^2) (**bottom**) of total Modeled PM2.5 for each microenvironment (home in, home out, work in, work out, other in, other out, in vehicle) for each participant. Results are sorted by median values of the total 24-hour daily dose from highest to lowest as shown in Figure 2. Shown are medians with 25th and 75th percentiles, and whiskers for minimum and maximum values.

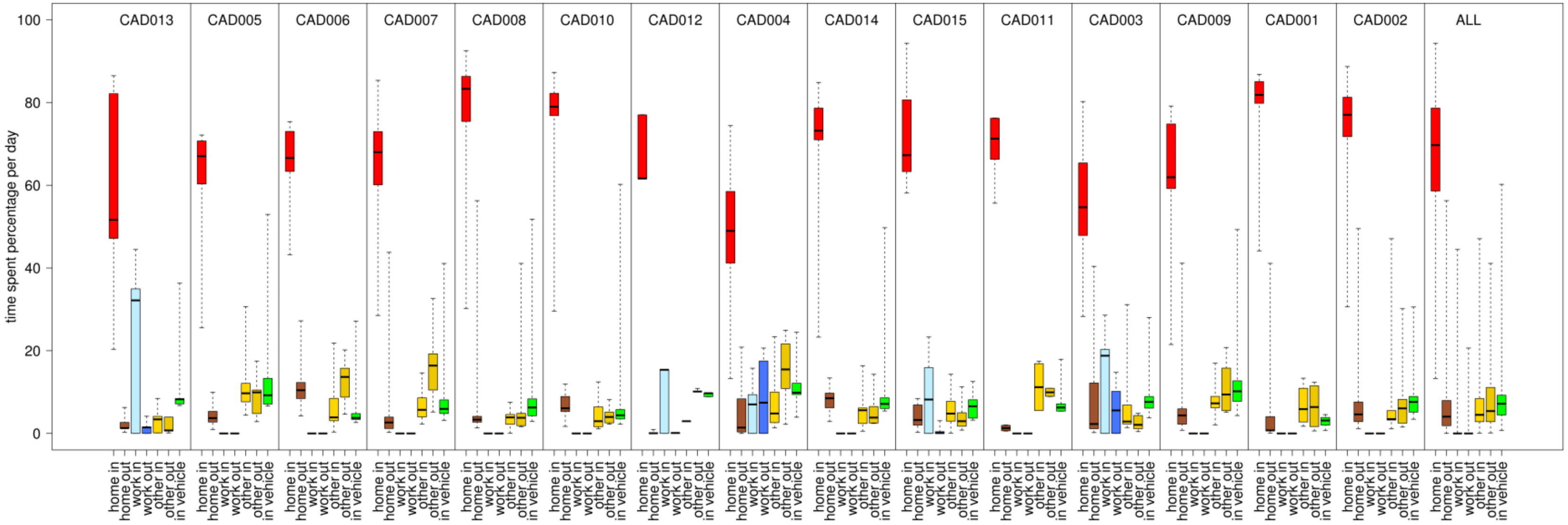
CAD013
CAD005
CAD006
CAD007
CAD008
CAD010
CAD012
CAD004
CAD014
CAD015
CAD011
CAD003
CAD009
CAD001
CAD002
ALL
time spent percentage per day
home in
home out
work in
work out
other in
other out
in vehicle

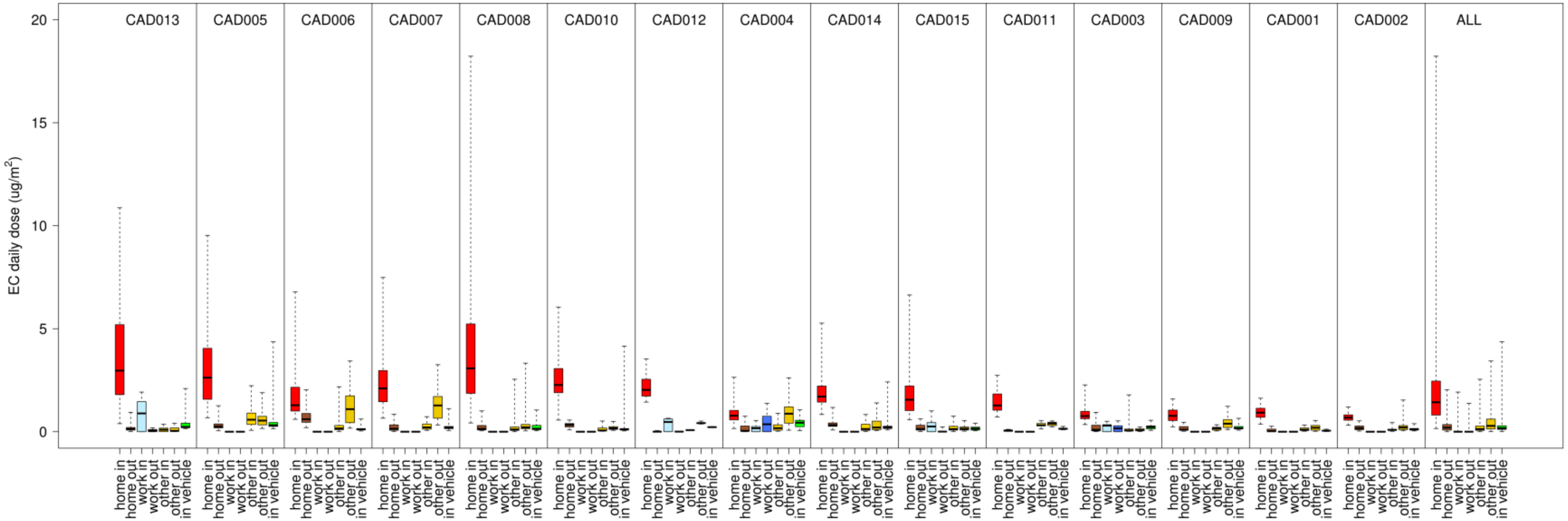

Figure S2. Percentage of time spent per day(%) (**top**) and daily dose (ug/m²) (**bottom**) of total Modeled EC for each microenvironment (home in, home out, work in, work out, other in, other out, in vehicle) for each participant. Results are sorted by median values of the total 24-hour daily dose from highest to lowest as shown in Figure 2. Shown are medians with 25th and 75th percentiles, and whiskers for minimum and maximum values.

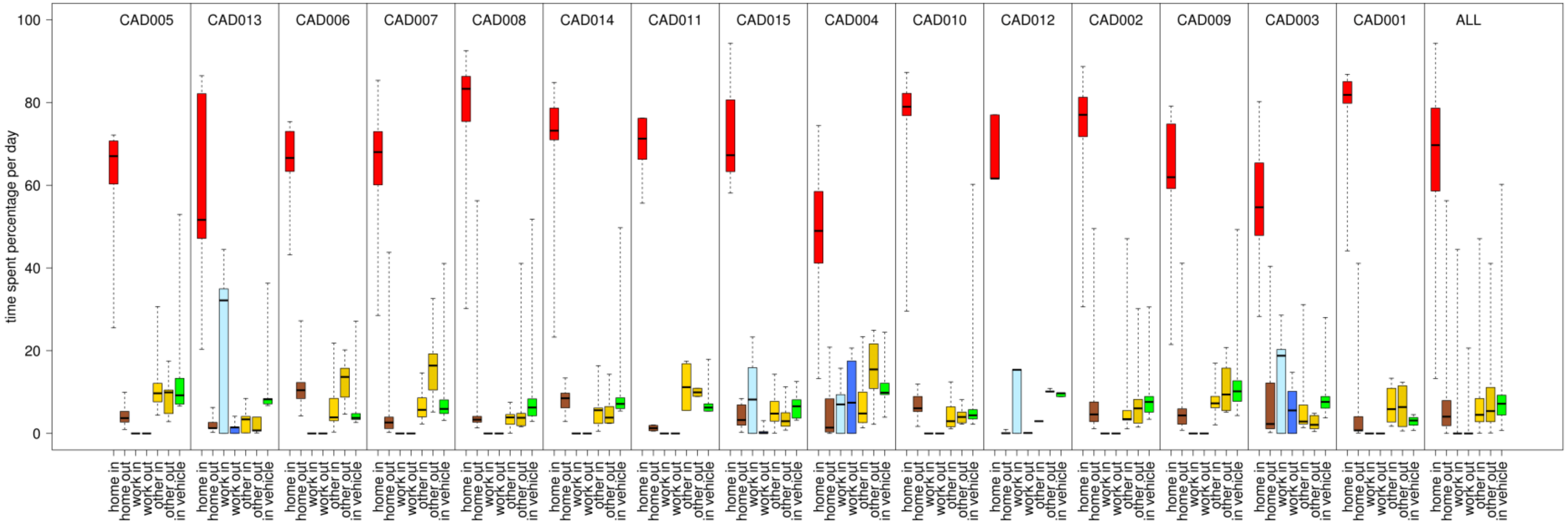
CAD005
CAD013
CAD006
CAD007
CAD008
CAD014
CAD011
CAD015
CAD004
CAD010
CAD012
CAD002
CAD009
CAD003
CAD001
ALL
time spent percentage per day
0
20
40
60
80
100
home in
home out
work in
work out
other in
other out
in vehicle

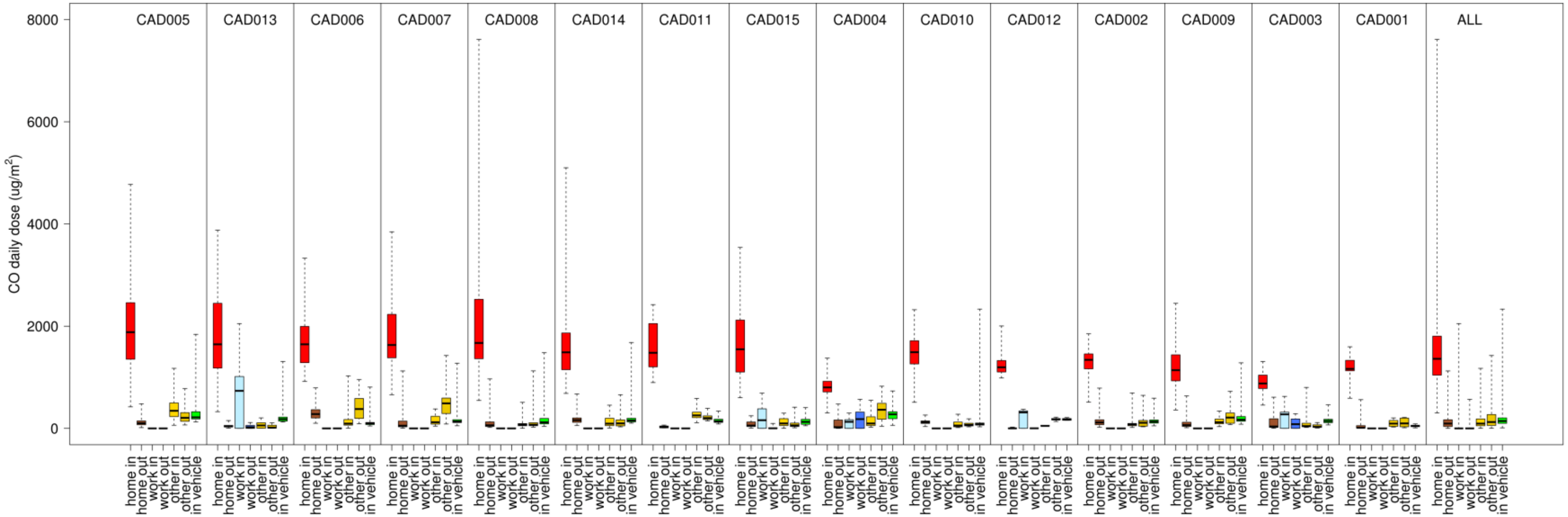

Figure S3. Percentage of time spent per day(%) (top) and daily dose (ug/m²) (bottom) of total Modeled CO for each microenvironment (home in, home out, work in, work out, other in, other out, in vehicle) for each participant. Results are sorted by median values of the total 24-hour daily dose from highest to lowest as shown in Figure 2. Shown are medians with 25th and 75th percentiles, and whiskers for minimum and maximum values.

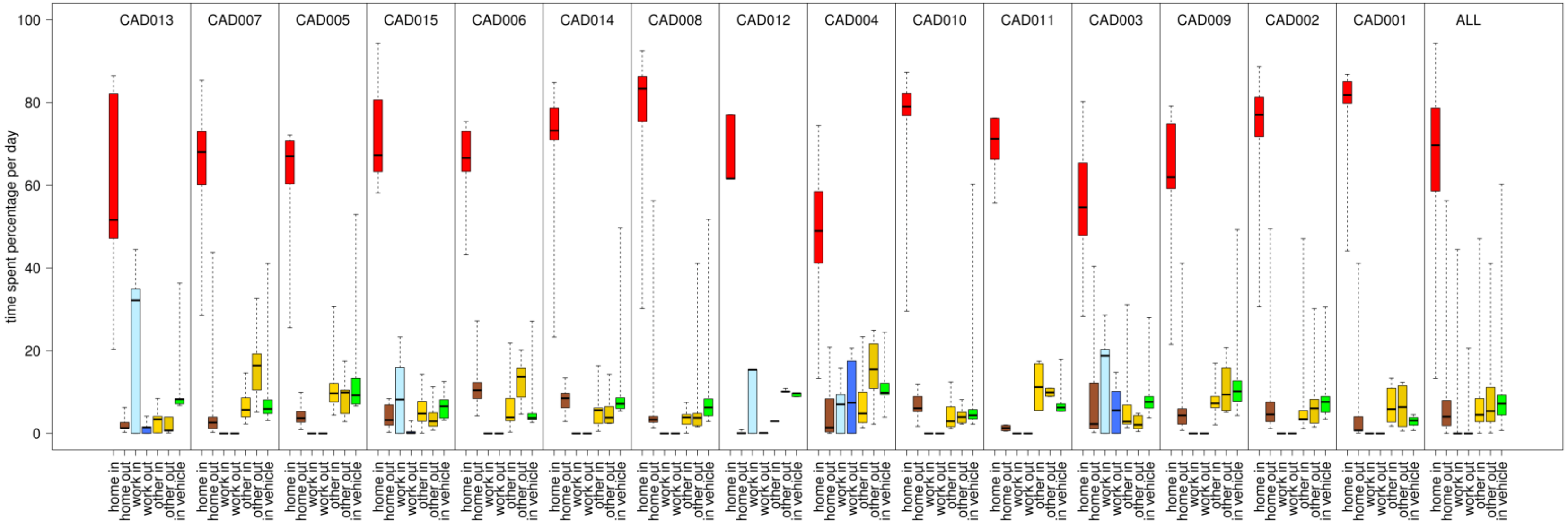
CAD013
CAD007
CAD005
CAD015
CAD006
CAD014
CAD008
CAD012
CAD004
CAD010
CAD011
CAD003
CAD009
CAD002
CAD001
ALL
time spent percentage per day
0
20
40
60
80
100
home in
home out
work in
work out
other in
other out
in vehicle

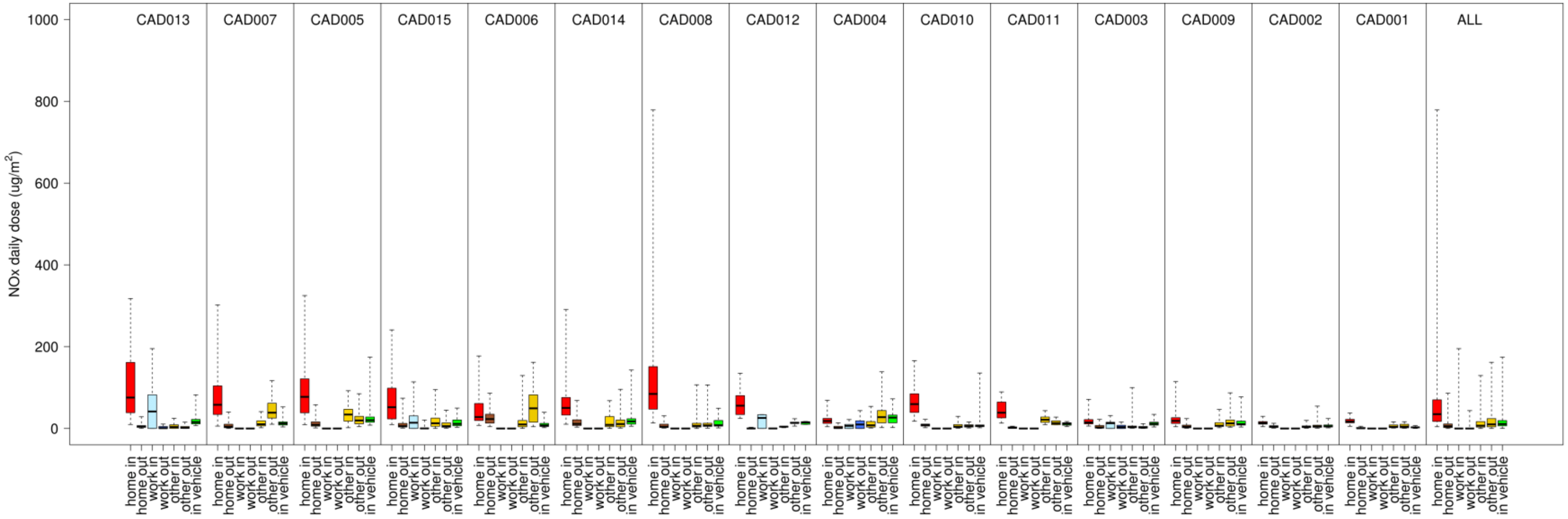

Figure S4. Percentage of time spent per day (%) (**top**) and daily dose (ug/m^2) (**bottom**) of total Modeled NOx for each microenvironment (home in, home out, work in, work out, other in, other out, in vehicle) for each participant. Results are sorted by median values of the total 24-hour daily dose from highest to lowest as shown in Figure 2. Shown are medians with 25th and 75th percentiles, and whiskers for minimum and maximum values.

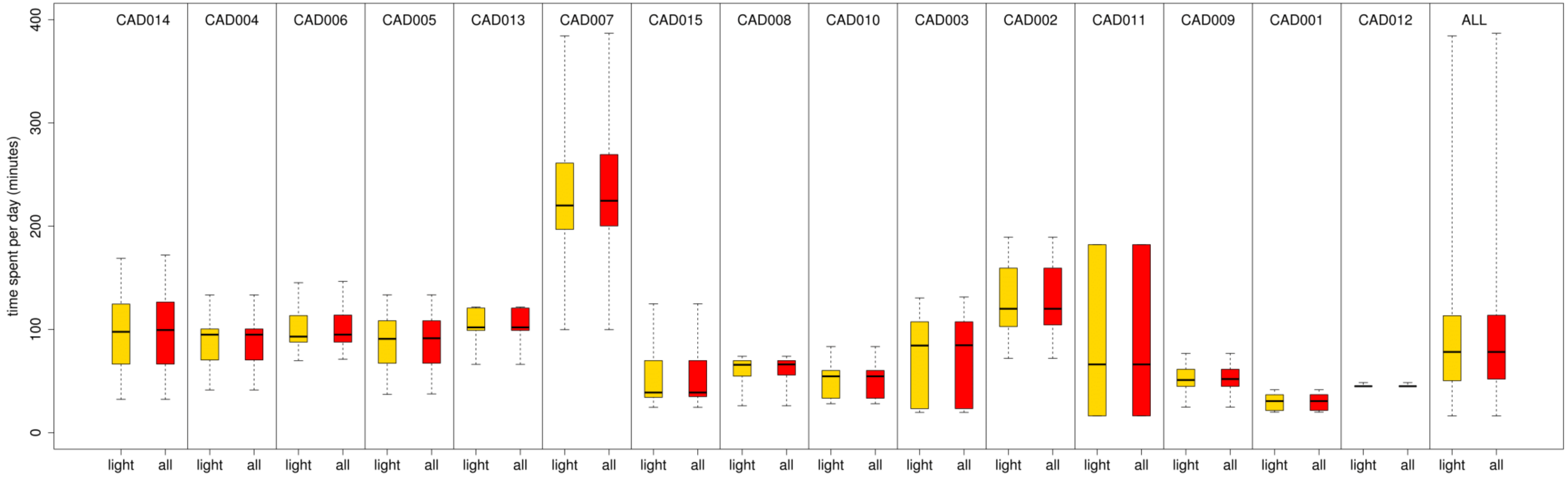

Figure S5. Time spent per day (minutes) at different activity levels (light activity and all activities including light, moderate and vigorous) for each participant. Results are sorted by median values of the total 24-hour PM2.5 daily dose from highest to lowest as shown in Figure 2. Shown are medians with 25th and 75th percentiles, and whiskers for minimum and maximum values.

References

1 Breen, M.S.; Breen, M.; Williams, R.W.; Schultz, B.D. Predicting residential air exchange rates from questionnaires and meteorology: model evaluation in central North Carolina. *Environ Sci Technol*, **2010**, *44*, 9349–9356.

2 Chan, W.R.; Nazaroff, W.W.; Price, P.N.; Sohn, M.D.; Gadgil, A.J. Analyzing a database of residential air leakage in the United States. *Atmos Environ* **2005**, *39*, 3445–3455.

3 Breen, M.S.; Seppanen, C.; Isakov, V.; Arunachalam, S.; Breen, M.; Samet, J.; Tong, H. Development of TracMyAir smartphone application for modeling exposures to ambient PM2.5 and ozone. *Int J Environ Res Public Health* **2019**, *16*, doi:10.3390/ijerph16183468.

4 Breen, M.S.; Yadong, X.; Williams, R.; Schneider, A.; Devlin, R. Modeling Individual-level Exposures to Ambient PM2.5 for the Diabetes and the Environment Panel Study (DEPS). *Sci. Total Environ.* **2018,** *626*, 807-816; https://doi.org/10.1016/j.scitotenv.2018.01.139

5 Breen, M.S.; Long, T.C.; Schultz; B.D.; Williams, R.W.; Richmond-Bryant, J.; Breen, M.; Langstaff, J.E.; Devlin, R.B.; Schneider, A.; Burke, J.M.; Batterman, S.A.; Meng, Q.Y. Air Pollution Exposure Model for Individuals (EMI) in Health Studies: Evaluation for Ambient $PM_{2.5}$ in Central North Carolina. *Environmental Science & Technology*, **2015**, *49*, 14184-14194; https://doi.org/10.1021/acs.est.5b02765

6 Meng, Q.Y.; Turpin, B.J.; Polidori, A.; Lee, J.H.; Weisel, C.; Morandi, M.; Colome, S.; Stock, T.; Winer, A.; Zhang, J. PM2.5 of ambient origin: Estimates and exposure errors relevant to PM epidemiology. *Environmental Science & Technology*, **2005**, *39*, 5105–5112; https://doi.org/10.1021/es048226f

7 Weschler, C.J.; Shields, H.C.; Nalk, D.V. Indoor Chemistry Involving O-3, No, and No2 as Evidenced by 14 Months of Measurements at a Site in Southern California. *Environmental Science & Technology*, **1994**, 28(12), 2120–2132; https://doi.org/10.1021/es00061a021

8 Dionisio, K.L.; Baxter, L.K.; Chang, H.H. An empirical assessment of exposure measurement error and effect attenuation in bipollutant epidemiologic models. *Environmental health perspectives*, **2014**, *122*, 1216–1224; https://doi.org/10.1289/ehp.1307772

9 Burke, J.M.; Zufall, M.J.; Ozkaynak, H. A population exposure model for particulate matter: case study results for PM2.5 in Philadelphia, PA. *J Expo Anal Environ Epidemiol*. **2001**, *11*, 470–489; https://doi.org/10.1038/sj.jea.7500188

10 Ott, W.; Klepeis, N.; Switzer, P. Air change rates of motor vehicles and in-vehicle pollutant concentrations from secondhand smoke. *J Expo Anal Environ Epidemiol*., **2008**; *18*, 312–325; https://doi.org/10.1038/sj.jes.7500601

11 The 2009 ASHRAE Handbook-Fundamentals, American Society of Heating, Refrigerating, and Air Conditioning Engineers: Atlanta, GA, 2009.
